# Recent advancements in multimodal human–robot interaction

**DOI:** 10.3389/fnbot.2023.1084000

**Published:** 2023-05-11

**Authors:** Hang Su, Wen Qi, Jiahao Chen, Chenguang Yang, Juan Sandoval, Med Amine Laribi

**Affiliations:** ^1^Department of Electronics, Information and Bioengineering, Politecnico di Milano, Milan, Italy; ^2^School of Future Technology, South China University of Technology, Guangzhou, China; ^3^State Key Laboratory of Management and Control for Complex Systems, Institute of Automation, Chinese Academy of Sciences, Beijing, China; ^4^Bristol Robotics Laboratory, University of the West of England, Bristol, United Kingdom; ^5^Department of GMSC, Pprime Institute, CNRS, ENSMA, University of Poitiers, Poitiers, France

**Keywords:** multi-modal signal processing, multi-modal feedback, multi-modal human–robot interaction, physical human–robot interaction, human–robot interaction

## Abstract

Robotics have advanced significantly over the years, and human–robot interaction (HRI) is now playing an important role in delivering the best user experience, cutting down on laborious tasks, and raising public acceptance of robots. New HRI approaches are necessary to promote the evolution of robots, with a more natural and flexible interaction manner clearly the most crucial. As a newly emerging approach to HRI, multimodal HRI is a method for individuals to communicate with a robot using various modalities, including voice, image, text, eye movement, and touch, as well as bio-signals like EEG and ECG. It is a broad field closely related to cognitive science, ergonomics, multimedia technology, and virtual reality, with numerous applications springing up each year. However, little research has been done to summarize the current development and future trend of HRI. To this end, this paper systematically reviews the state of the art of multimodal HRI on its applications by summing up the latest research articles relevant to this field. Moreover, the research development in terms of the input signal and the output signal is also covered in this manuscript.

## 1. Introduction

Recent years have witnessed a huge leap in the advancement of robotics, yet it is quite challenging to build a robot that can communicate with individuals naturally and synthesize understandable multimodal motions in a variety of interaction scenarios. To deliver appropriate feedback, the robot requires a high level of multimodal recognition in order to comprehend the person's inner moods, goals, and character. Devices for human–robot interaction (HRI) have become a common part of everyday life thanks to the growth of the Internet of Things. The input and output of a single sense modality, such as sight, touch, sound, scent, or flavor, is no longer the only option for HRI.

**Figure 1:**
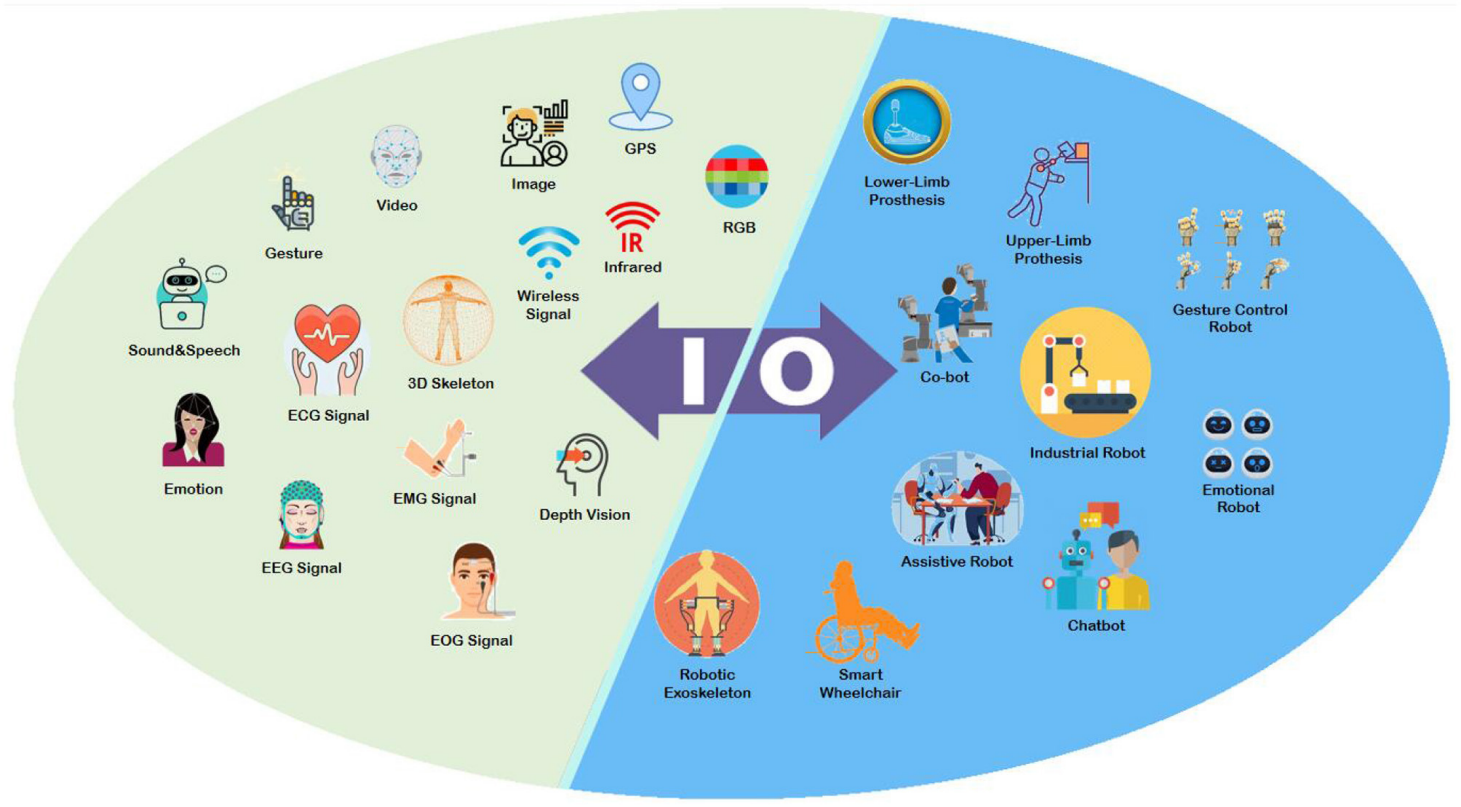
The various signals of multimodal HRI.

The goal of multimodal HRI is to communicate with a robot utilizing various multimodal signals (Figure 1), including voice, image, text, eye movement, and touch. Multimodal HRI is a broad field that is closely associated with cognitive science, ergonomics, communication technologies, and virtual reality. It includes both multimodal input signals from humans to robots and multimodal output signals from robots to humans. As the carrier of the Internet of Things in the era of big data, multimodal HRI is closely connected to the advancement of visual effects, AI, sentimental data processing, psychological and physiological appraisal, distance education, as well as medical rehabilitative services.

The earliest studies on multimodal HRI date back to the 1990s, and several publications offer an interactive approach that combines voice and gesture. Furthermore, the rise of immersive visualization opens up a new multimodal interactive interface for HRI: an immersive world that blends visual, aural, tactile, and other sense modalities. Immersive visualization, which integrates multimodal channels and multimodalities, has become an inseparable part of high-dimensional big data visualization.

Great strides have been made in robotics over the past years, with human-computer interaction technology playing a critical role in improving the user experience, reducing tiresome processes, and promoting the acceptance of robots. Novel human-computer interaction strategies are necessary to further robotics progress, and a more natural and adaptable interaction style is particularly important (Fang et al., 2019). In many application areas, robots must process output signals in the same way as human beings. Visual and auditory signals are the most straightforward methods for individuals to interact with home robots. With the advancement of statistical modeling, speech recognition has been increasingly employed in robotics and smart gadgets to enable natural language-based HRI. Furthermore, significant progress in picture recognition has been made (Xie et al., 2020), with some robots able to comprehend instructions given to them in human language and perform necessary activities by combining visual and aural input signals.

**Figure 2:**
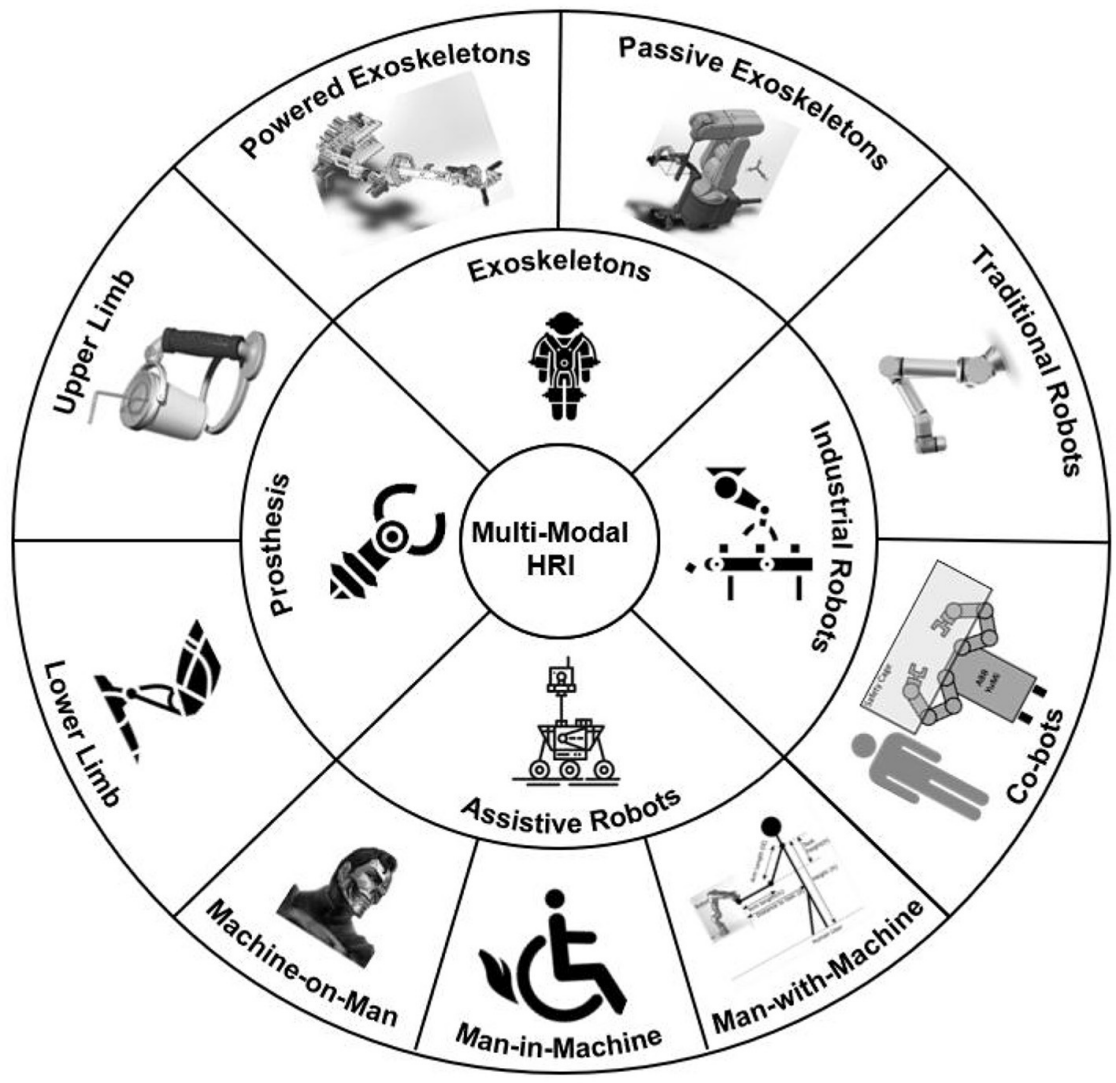
Major application areas of multimodal HRI (Manna and Bhaumik, 2013; Klauer et al., 2014; Gopinathan et al., 2017; K'´ut'´uk et al., 2019).

This paper systematically follows the state of the art of multimodal HRI and thoroughly reviews the research progress in terms of the signal input, the signal output, and the applications of multimodal HRI (Figure 2). Specifically, this article elaborates on the research progress of signal input of multimodal HRI from three perspectives: gesture input and recognition, speech input and recognition, as well as emotion input and recognition. In terms of information output, gesture generation and emotional expression generation are covered. The latest applications of multimodal HRI, including assistive mobile robots, robotic exoskeletons, as well as robotic prostheses, will also be introduced.

## 2. Methodology

**Figure 3:**
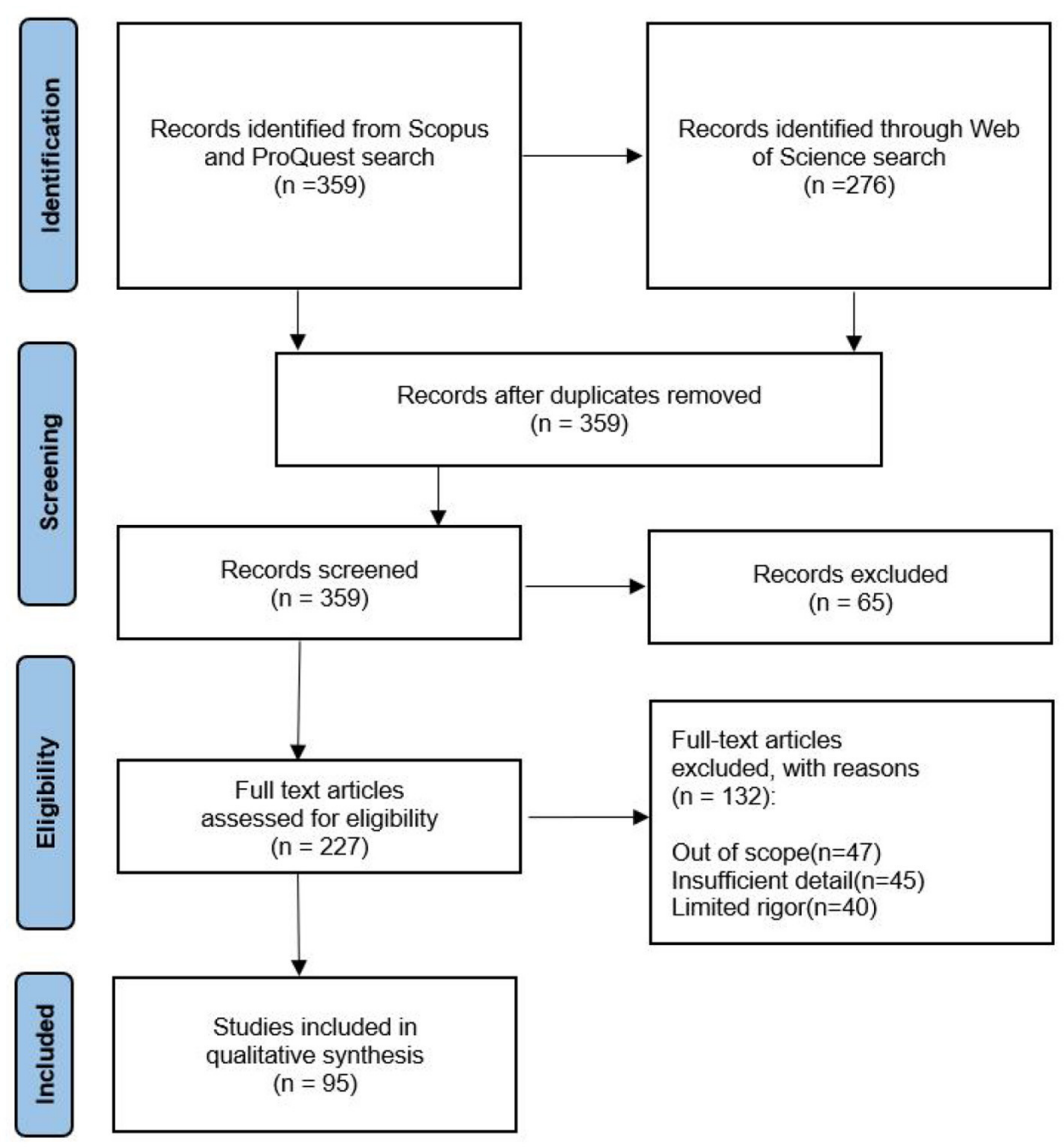
PRISMA flow diagram describing the search results and screening (Source: Processed by authors).

By using Preferred Reporting Items for Systematic Reviews and Meta-analysis (PRISMA) guidelines, a systematic review of recently published literature was conducted on recent advancements in multimodal human–robot Interaction (Page et al., 2021). The inclusion criteria were: (i) publications indexed in the Web of Science, Scopus, and ProQuest databases; (ii) publication dates between 2008 and 2022; (iii) written in English; (iv) being a review paper or an innovative empirical study; and (v) certain search terms covered. The exclusion criteria were: (i) editorial materials, (ii) conference proceedings, and (iii) books were removed from the research. The Systematic Review Data Repository (SRDR), a software program for the collection, processing, and inspection of data for our systematic review, was employed. The quality of the specified scholarly sources was evaluated by using the Mixed Method Appraisal Tool. After extracting and analyzing publicly accessible papers as evidence, no institutional ethics approval was required before starting our research (Figure 3).

**Figure 4:**
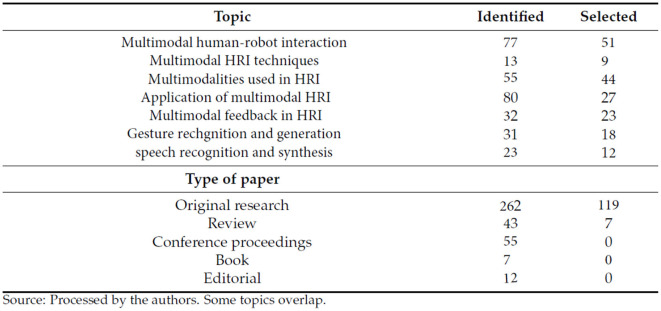
Topics and types of paper identified and selected.

Throughout April 2008 and October 2022 (mostly in 2022), a systematic literature review of the Web of Science, ProQuest, and Scopus databases was performed, with search terms including “multimodal human–robot interaction,” “multimodal HRI techniques,” “multimodalities used in HRI,” “speech recognition in HR,” “application of multimodal HRI,” “gesture recognition in HRI,” and “multimodal feedback in HRI.” The search keywords were determined as the most frequently used words or phrases in the researched literature. Because the examined research was published between 2008 and 2022, only 359 publications met the qualifying requirements. We chose 227 primarily empirical sources by excluding ambiguous or controversial findings (insufficient/irrelevant data), outcomes unsubstantiated by replication, excessively broad material, or having nearly identical titles (Figure 4).

## 3. Modalities used in human–robot interaction

There are several modalities that are currently used in human–robot interaction, including audio, visual, haptic, kinesthetic, and proprioceptive modality (Navarro et al., 2015; Li and Zhang, 2017; Ferlinc et al., 2019; Deuerlein et al., 2021; Groechel et al., 2021). These modalities can be used alone or in combination to enable different forms of human–robot interaction, such as voice commands, visual gestures, and physical touch. Additionally, some researchers work on improving the quality of interaction and the perceived “intelligence” of the robot by incorporating tools like natural language processing, cognitive architectures, and social signal processing.

### 3.1. Audio modality

The audio modality is an important aspect of human–robot interaction as it allows for verbal communication between humans and robots. In order for robots to effectively understand and respond to human speech, they must be equipped with speech recognition and natural language processing (NLP) capabilities.

Robots that use audio modalities can recognize and generate human speech through the use of speech recognition and synthesis technologies (Lackey et al., 2011; Luo et al., 2011; Zhao et al., 2012; Tsiami et al., 2018; Deuerlein et al., 2021). Speech recognition allows the robot to understand spoken commands or questions from a human, while speech synthesis allows the robot to generate spoken responses or instructions. This modality is used in several application such as voice assistants, voice-controlled robots, and even some language tutor robots (House et al., 2009; Belpaeme et al., 2018; Humphry and Chesher, 2021).

### 3.2. Visual modality

Visual modality allows the robot to perceive and interpret visual cues such as facial expressions, gestures, body language, and gaze direction. Robots that employ visual modalities can perceive their environment using cameras and process visual information using computer vision algorithms (Hasanuzzaman et al., 2007; Li and Zhang, 2017). These algorithms can be used to recognize objects, faces, and gestures, as well as to track the motion of humans and other objects. This modality is used in applications such as robot navigation, surveillance, and human–robot interaction.

Recent advances in computer vision and deep learning have led to significant improvements in the ability of robots to recognize and interpret visual cues, making them more effective in human–robot interactions (Celiktutan et al., 2018). One area of research in visual modality is the use of facial expression recognition, which enables a robot to understand a person's emotions and respond accordingly. This can make the interaction more natural and intuitive for the human. Another area of research is the use of gesture recognition, which allows a robot to understand and respond to human gestures, such as pointing or nodding. This can be useful in tasks such as navigation or object manipulation. In addition, visual saliency detection, which allows the robot to focus on the most important aspects of the visual scene, and object recognition, which enables the robot to identify and locate objects in the environment, are also important areas of research in visual modality.

### 3.3. Haptic modality

Haptic modality enables touch-based communication between humans and robots, including the robot's ability to sense and respond to touch and to apply force or vibrations to the human. Recent advances in haptic technology have led to the development of more advanced haptic interfaces, such as force feedback devices and tactile sensors (Navarro et al., 2015; Pyo et al., 2021). These devices allow robots to provide a wider range of haptic cues, which can be used in applications such as robotic surgery, prosthetics, and tactile communication. One area of research in haptic modality is the use of force feedback, which allows a robot to apply forces to a person, making the interaction more natural and intuitive. Another area of research is the use of tactile sensing, which allows a robot to sense the texture, shape, and temperature of objects, and to respond accordingly.

### 3.4. Kinesthetic modality

The kinesthetic modality is an aspect of human–robot interaction that relates to the ability of the robot to sense and respond to motion and movement. This includes the ability of the robot to sense and respond to the motion of the human body, such as posture, gait, and joint angles. Robots that use kinesthetic modalities can sense and control their own movement. This can be done by using sensors to measure the position and movement of the robot's joints, and actuators to control those joints (Groechel et al., 2021). This modality is used in applications such as industrial robots, bipedal robots, and robots for search and rescue.

### 3.5. Proprioceptive modality

Proprioception refers to the ability of an organism to sense the position, orientation, and movement of its own body parts (Ferlinc et al., 2019). In human–robot interaction, proprioception can be used to allow robots to sense and respond to the position and movement of their own body parts in relation to the environment and the human. Robots that use proprioceptive modalities can sense their internal state (Hoffman and Breazeal, 2008). This can include, for example, the position of their joints and the forces acting on their body. This information can be used to control the robot's movements, to detect and diagnose failures, and to plan its actions. For example, Malinovská et al. (2022) have developed a neural network model that can learn proprioceptive-tactile representations on a simulated humanoid robot, demonstrating the ability to accurately predict touch and its location from proprioceptive information. However, further work is needed to address the model's limitations.

All these modalities can be combined in different ways to provide robots with a wide range of capabilities and enhance their ability to interact with humans in natural ways. Additionally, for better human robot interaction, using modalities that are congruent with human communication, like visual and auditory modality, are preferred as it makes the interaction more intuitive and easy for human participants.

## 4. Techniques for multimodal human–robot interaction

**Figure 5:**
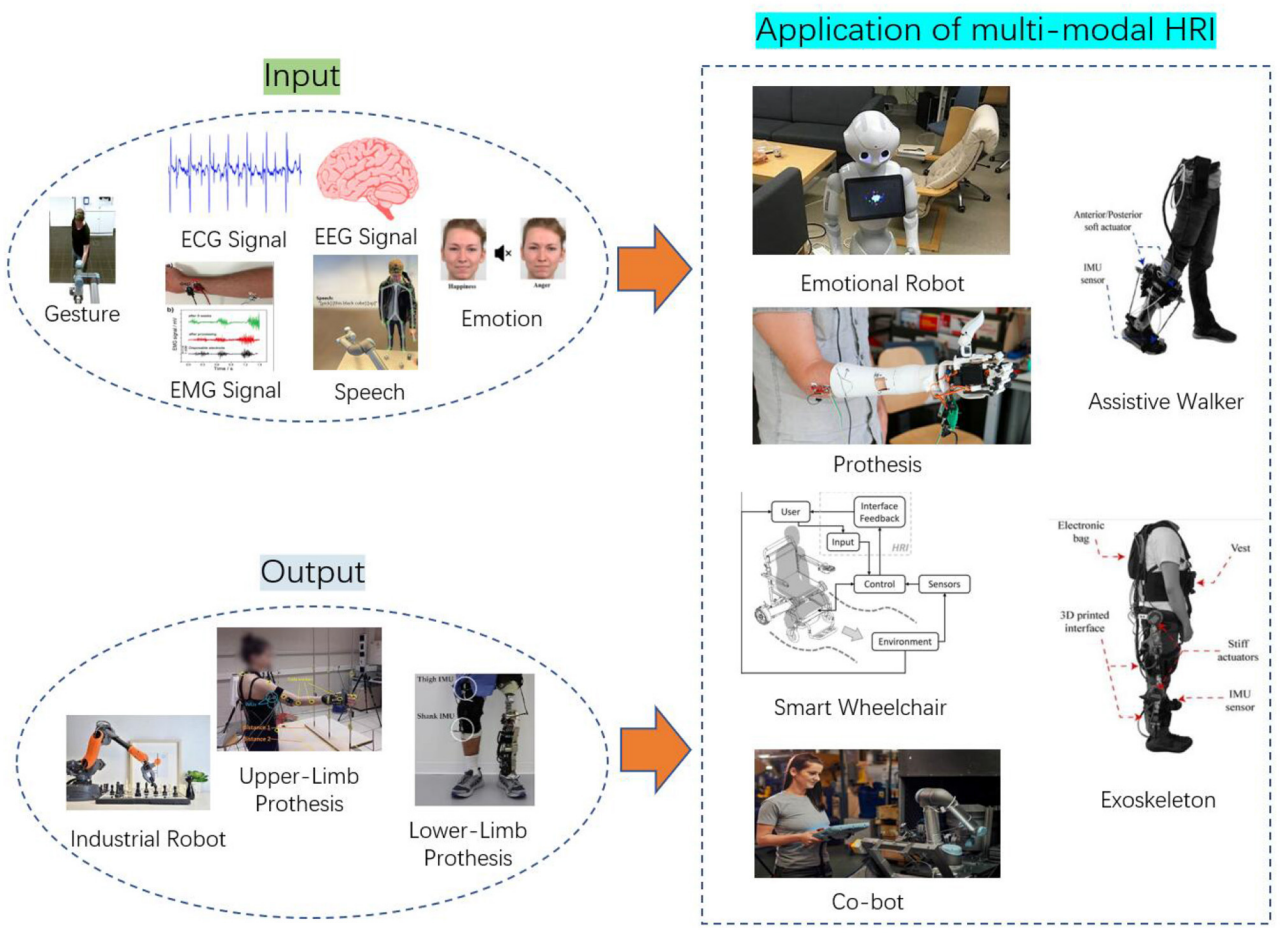
Multimodal HRI (Castillo et al., 2013; Yu et al., 2016; Lannoy et al., 2017; Legrand et al., 2018; Stephens-Fripp et al., 2018; Mohebbi, 2020; Khalifa et al., 2022; Otálora et al., 2022; Strazdas et al., 2022).

In multimodal HRI, social robots frequently use multimodal interaction methods comparable to those utilized by individuals: speech generation (through speakers), voice recognition (through microphones), gesture creation (through physical embodiment), and gesture recognition (via cameras or motion trackers) (Mead and Matarić, 2017). This section will provide a brief review on the signal input, signal output, as well as the practical application of multimodal human–robot interaction (Figure 5).

### 4.1. Multimodal signal processing for human–robot interaction

The last decade has seen great advancement in human–robot interaction. Nowadays, with more sophisticated and intelligent sensors, speech, gestures, images, videos, as well as physiological signals like electroencephalography (EEG) and electrocardiogram (ECG), can be input into robots and recognized by them. A brief introduction to the most common input signals employed in HRI will be given in this section.

#### 4.1.1. Computer vision

Robots equipped with cameras can recognize and track human faces and movements, allowing them to respond to visual cues and gestures (Andhare and Rawat, 2016; Maroto-Gómez et al., 2023). It is an important modality in multimodal human–robot interaction (HRI) as it allows robots to perceive and understand their environment and the actions of humans.

Object detection and tracking: Robots can use computer vision to detect and track objects and people in their environment (Redmon et al., 2016). This can be used for tasks such as following a person, avoiding obstacles, or manipulating objects.

Facial recognition: Robots can use computer vision to recognize and identify specific individuals by analyzing their facial features (Schroff et al., 2015). This can be used for tasks such as personalization, security, or tracking attendance.

Gesture recognition: Robots can use computer vision to recognize and interpret human hand and body gestures (Mitra and Acharya, 2007). This can be used as an additional modality for controlling the robot or issuing commands, rather than using speech or buttons, which is particularly useful in noisy environments.

Face and body language: Robots can use computer vision to detect and interpret facial expressions and body language, which can be used to infer the emotions or intent of a human, and generate appropriate responses, which is known as affective computing (Pantic and Rothkrantz, 2000).

Gaze tracking: Robots can use computer vision to track the gaze of a human in order to understand where their attention is focused (Smith et al., 2013). This can be used to infer human attention and interest or anticipate the next action, for example, a robot assistant can know that the human is going to pick an object by following their gaze.

Multi-Camera system: Using multiple cameras can enable a robot to track and understand the 3D space and provide more robust performance, such as enabling robots to walk without colliding with obstacles (Heikkila, 2000).

These are just a few examples of how computer vision can be used in HRI, and many other applications are being developed and explored in the field. Computer vision systems can be integrated with other modalities, such as speech recognition or haptic feedback, to create a more comprehensive HRI experience.

It's important to note, however, that computer vision can be a challenging technology to implement, especially when it comes to dealing with variations in lighting, occlusion, or viewpoint. It also could be affected by the environment, such as reflections, glare, or shadows, that can make it difficult for the robot to accurately interpret visual data (Tian et al., 2020).

#### 4.1.2. Natural language processing

Natural language processing (NLP) is an important modality in multimodal human–robot interaction (HRI) as it allows robots to understand and respond to human speech in a way that is more natural and intuitive (Scalise et al., 2018). Here are a few ways that NLP can be used in HRI.

Speech recognition: NLP can be used to convert speech into text, which can then be used to interpret human commands, queries, or requests. This allows the robot to understand and respond to spoken commands, such as “turn on the lights” or “navigate to the kitchen.”

Natural Language Understanding (NLU): After the speech is converted into text, the NLP system uses NLU to extract the intent and entities from the text (Kübler et al., 2011; Bastianelli et al., 2014). This allows the robot to understand the intent of the command and the objects or actions referred to by the entities, such as “set the temperature to 20 degrees” intent is “set” and “temperature” is the entities.

Natural Language Generation (NLG): Natural Language Generation (NLG) is a subfield of Natural language processing (NLP) that enables computers to produce natural language responses to humans. NLG has become an important aspect of human–robot interaction (HRI) due to its ability to allow robots to communicate with humans in a more human-like manner. However, the process of generating natural language responses involves retrieving and synthesizing relevant information from various data sources, such as open data repositories, domain-specific databases, and knowledge graphs. In addition, the NLG process involves the use of complex algorithms and statistical models to generate natural language responses that are contextually appropriate and grammatically correct.

Question answering: NLU and NLG together allows the robot to understand and generate answers to questions, for example “What's the weather today?”

Dialogue management: NLP can be used to manage the dialogue between the human and the robot, for example to track the state of the conversation, and allow for more seamless interactions, for example by remembering the context of the previous turns in the conversation and using it to generate appropriate responses.

Language translation: NLP can be used to translate text from one language to another, which can enable robots to interact with people who speak different languages.

NLP can be a challenging technology to implement, especially when it comes to handling variations in accent, dialect, or speech patterns, also, NLP models rely heavily on the training data, and thus the performance may not be accurate when it comes to handling new or unseen words, entities or concepts (Khurana et al., 2022).

#### 4.1.3. Gesture recognition

Gesture identification is a critical step in gesture recognition after unprocessed signals from sensors are obtained. Gesture identification is the discovery of gestural signals in raw data and the separation of the relevant gestural inputs. Popular solutions for solving the issue of gesture recognition are grounded in visual features, ML algorithms, and skeletal models (Mitra and Acharya, 2007; Rautaray and Agrawal, 2015). When it comes to detecting body gestures, the comprehensive representation of the body is ineffective from time to time. In contrast to the preceding methodologies, the skeleton model methodology uses a human skeleton to discover human body positions. The skeletal model technique is also advantageous for categorizing gestures. With the benefits listed above, the skeletal model method has emerged as an appealing solution for sensing devices (Mitra and Acharya, 2007; Cheng et al., 2015).

Among alternative communication modalities for human–robot and inter-robot interaction, hand gesture recognition is mostly employed. Hand gesture recognition can be divided into two categories: static hand gestures and dynamic hand gestures. Static hand gestures refer to specific hand postures or shapes that convey meaning without the need for movement. These gestures can be simple, such as a thumbs-up or a peace sign, or more complex, such as those used in sign languages for the deaf community. Static hand gestures have their advantages in specific contexts, such as low computational complexity and less dependency on temporal information.

In comparison to static hand gestures, the dynamic hand gestures of robots are more humanoid. Dynamic hand gestures are particularly versatile since the robotic hand may move in any direction and bend at practically any angle in all available coordinates; static hand gestures, on the other hand, are constrained to much fewer movements (Rautaray and Agrawal, 2015). A wide range of applications, involving smart homes, video surveillance, sign language recognition, human–robot interaction, and health care, have recently embraced dynamic hand gestures. All of these applications require high levels of accuracy against a busy background, optimum recognition, and temporal precision (Huenerfauth and Lu, 2014; Ur Rehman et al., 2022).

#### 4.1.4. Emotion recognition

Emotions are inherent human characteristics that impact choices along with behaviors, and they are crucial in interaction and emotional intelligence (Salovey and Mayer, 2004), i.e., the capacity to comprehend, utilize, and command feelings, is substantial for effective relationships. Affective computation seeks to provide robots with emotional intelligence, aiming to improve natural human–robot interaction. Humanoid competencies of observation, comprehension, and feeling output are sought in the context of human–robot interaction. Emotions in HRI can be examined from three distinct perspectives, as follows.

Formalization of the robot's internal psychological state: Adding sentimental characteristics to individuals and bots can increase their efficacy, adaptability, and plausibility. In recent years, robots have been produced to mimic feelings by determining neurocomputational frameworks, formalizing them in pre-existing cognitive architectures, modifying well-known mental representations, or developing specific affective designs (Saunderson and Nejat, 2019).

The emotional response of robots: The capacity of robots to display recognizable emotional responses has a significant influence on human–robot interaction in complicated communication scenarios (Rossi et al., 2020). Numerous research examined how individuals perceive and identify sentimental reactions through modalities (postures, expressions, motions, and voices) might transmit emotional signals from robots to humans.

Robotic applications that can detect and comprehend human feelings are competent in social interactions. Recent research aims to develop algorithms for categorizing psychological states from many input signals, including speech, body language, expression, and physiological signals (Cavallo et al., 2018).

Furthermore, sentiment identification is a multidisciplinary area that necessitates expertise from a variety of disciplines, including psychological science, neurology, data processing, electronics, and AI. It may be handled using multimodal signals, including physiological signals like EEG, GSR, or heart rate fluctuations measured by BVP or EKG. As with BVP and GSR, these are inner signals that represent the equilibrium of the parasympathetic and sympathetic nervous systems, whereas EEG shows variations in the cortex parts of the brain (Das et al., 2016). Externally visible indications, on the other hand, include facial expressions, bodily motions, and voice. While internal signals are thought to be more impartial due to the inherent qualities of several operational parts of the central nervous system, external signals remain subjective measures of expressed feelings (Yao, 2016).

### 4.2. Multimodal feedback for human–robot interaction

Developing a socially competent robot capable of interacting naturally with individuals and synthesizing adequately intelligible multimodal actions in a wide range of interaction scenarios is a difficult task. This necessitates a high degree of multimodal perception of robots, since they must comprehend the human's mental moods, goals, and character aspects in order to provide proper feedback.

#### 4.2.1. Speech synthesis

Speech synthesis, also known as text-to-speech (TTS), is an important approach in multimodal human–robot interaction (HRI) as it allows robots to provide verbal feedback or instructions to the human user in a way that is similar to how a human would (Luo et al., 2011; Ashok et al., 2022). Robots can use text-to-speech (TTS) technology to generate spoken responses to humans. Here is how speech synthesis works in more detail:

1. Text is generated by the robot's onboard computer in response to a user request, or based on data the robot needs to communicate. However, the generation of text to be uttered (Natural Language Generation) is a research field. On a superficial level, responses can either be template-based (i.e., scripted by humans), retrieved from knowledge sources (typically, the Internet) or generated using large-language models.
2. Once the text has been generated, it is passed to a Text-to-Speech (TTS) engine, which uses a set of rules, or a machine learning model, to convert the text into speech. This process involves transforming the written text into a phonetic representation that can be pronounced by the robot.
3. The TTS engine can be tuned to mimic different human voices, genders, and even create a virtual robot voice. For example, many systems make use of widely available TTS engines (e.g., Acapela, Cereproc, Google TTS), which offer a range of voices in different languages and accents.
4. The generated speech is then output through the robot's speakers, allowing the user to hear the response. The quality of the output speech is dependent on the TTS engine, the quality of the audio hardware, and the environmental conditions in which the robot is operating.
5. The output can be in different languages, depending on the specific application and the need. For instance, some robots may be designed to operate in multilingual environments and require the ability to speak multiple languages to communicate effectively with users.

Speech synthesis can be integrated with other modalities, such as computer vision, or natural language processing (NLP), to create a more comprehensive HRI experience. For example, a robot that uses speech recognition and NLP to understand spoken commands can use speech synthesis to provide verbal feedback, such as “I'm sorry, I didn't understand that command.”

Speech synthesis can also be used to provide instructions, such as “Please put the object on the tray,” or to answer questions, such as “The current temperature is 20 degrees.”

Speech synthesis can enhance the user experience by making the interaction with the robot more natural, intuitive, and engaging. It can also be used to provide information or instructions in a variety of languages, making the robot accessible to a wider range of users.

#### 4.2.2. Visual feedback

Visual feedback is an important output modality in multimodal human–robot interaction (HRI) as it allows robots to provide feedback to the human user through visual cues (Gams and Ude, 2016; Yoon et al., 2017). Here are a few ways that visual feedback can be used in HRI.

Status indication: Robots can use lights, displays, or other visual cues to indicate the status of the robot, such as when the robot is ready to receive commands, when it is performing a task, or when it has completed a task (Admoni and Scassellati, 2016).

Error indication: Robots can use visual cues such as flashing lights or error messages to indicate an error or problem with the robot, for example when the robot can't complete a task due to an obstacle or error (Kim et al., 2016).

Wayfinding: Robots can use visual cues such as arrows or maps, to indicate a path or a location, this can help the user to navigate and orient themselves in the environment (Giudice and Legge, 2008).

Object recognition and tracking: Robots can use visual cues such as highlighted boxes, to indicate the objects or areas of interest the robot is tracking or recognizing (Cazzato et al., 2020).

Expressions and emotions: Robots can use visual cues such as facial expressions or body language, to indicate the robot's emotions or intent, similar to the way humans communicate non-verbally (Al-Nafjan et al., 2017).

Multi-Camera Systems: Robots can use multiple cameras to provide visual feedback, by showing multiple views of the environment, or provide 3D information, which can help the user to understand the robot's perception of the environment (Feng et al., 2017).

Displaying conversation: In addition to the above, visual feedback can also be used to display the content of the conversation in HRI, such as what the robot “hears” through automatic speech recognition (ASR) and what it is saying. This can be particularly useful for individuals with hearing impairments or in noisy environments where auditory feedback may not be sufficient (Rasouli et al., 2018).

#### 4.2.3. Visual feedback

Visual feedback is an important output modality in multimodal human–robot interaction (HRI) as it allows robots to provide feedback to the human user through visual cues (Gams and Ude, 2016; Yoon et al., 2017). Here are a few ways that visual feedback can be used in HRI.

Status indication: Robots can use lights, displays, or other visual cues to indicate the status of the robot, such as when the robot is ready to receive commands, when it is performing a task, or when it has completed a task.

Error indication: Robots can use visual cues such as flashing lights or error messages to indicate an error or problem with the robot, for example when the robot can't complete a task due to an obstacle or error.

Wayfinding: Robots can use visual cues such as arrows or maps, to indicate a path or a location, this can help the user to navigate and orient themselves in the environment.

Object recognition and tracking: Robots can use visual cues such as highlighted boxes, to indicate the objects or areas of interest the robot is tracking or recognizing.

Expressions and emotions: Robots can use visual cues such as facial expressions or body language, to indicate the robot's emotions or intent, similar to the way humans communicate non-verbally.

Multi-Camera Systems: Robots can use multiple cameras to provide visual feedback, by showing multiple views of the environment, or provide 3D information, which can help the user to understand the robot's perception of the environment.

Displaying conversation: In addition to the above, visual feedback can also be used to display the content of the conversation in HRI, such as what the robot “hears” through ASR (automatic speech recognition) and what it is saying. This can be particularly useful for individuals with hearing impairments or in noisy environments where auditory feedback may not be sufficient.

#### 4.2.4. Gesture generation

In general, gesture generation is an area that remains largely underdeveloped in robotics research, with most of the focus being on gesture recognition. In conventional robotics, recognition always predominates over gesture synthesis. The term “gesture” has been commonly utilized to refer to item manipulation tasks instead of non-verbal expressive behaviors among the few extant systems that are really devoted to gesture synthesizing. Computational techniques to synthesize multimodal action may be divided into three steps: identifying what to express, deciding how to transmit it, and lastly, acting on it (Covington, 2001). Although the Articulated Communicator Engine acts at the behavioral realization layer, the entire system employed by the digital assistant Max consists of a combined content and behavioral planning architecture (Kopp et al., 2008).

Utilizing multimodal Utterance Representation Markup Language, gesture expressions inside the Articulated Communicator Engine (ACE) framework may be defined in two distinct ways (Salem et al., 2012). A gesture's exterior representation, such as the posture of the gesture stroke, can be clearly articulated in verbal words and co-verbal gestures, which are classified as feature-based explanations (Gozzi et al., 2022). By correlating temporal markers, gesture association to certain language pieces is discovered. Secondly, gestures may be defined as keyframe animation, where each keyframe defines a “key posture,” a component of the general gesture motion that describes the condition of each joint at that particular moment. Assigned time IDs are used to gather speed data for the interpolation between every two key postures and the related association to portions of speech. In ACE, keyframe animations may be created manually or via motion-capture data from a human presenter, enabling real-time animation of virtual agents. Each pitch phrase and co-expressive gesture expression in a multimodal utterance reflects a single thought unit, often known as a chunk of speech-gesture production (Kopp and Wachsmuth, 2004).

The ACE engine uses the following timing for gestures online: The basic way to establish synchronicity within a chunk is to modify the gesture to match the pace and structure of speech. To this end, the ACE scheduler gets millisecond-level scheduling details about the synthesized voice and uses those details to determine the beginning and end of the gesture stroke. Each individual gesture component receives an automated propagation of these timing limitations (Kopp and Wachsmuth, 2004). Chae et al. (2022) developed a methodology that enables robots to generate co-speech gestures automatically, based on a morphemic analysis of the sentence of utterance. After determining the expression unit and the corresponding gesture type, a database of motion primitives is used to retrieve an appropriate gesture that conveys the robot's thoughts and feelings. The method showed promising results, with 83% accuracy in determining expression units and gesture types, and positive feedback from a user study with a humanoid robot.

#### 4.2.5. Emotional expression generation

In-home robot and service robot has received much attention recently, and the demand for service robots is expected to expand rapidly in the coming years. Human-centerd operations are among the most intriguing aspects of smart service robots. Smart interaction is an important characteristic of service robots in care services, companionship, and entertainment. In real-world settings, emotional intelligence will be critical for a robot to participate in an amicable conversation. Furthermore, there has been a surge in interest in researching robotic mood-generating methods that aim to offer a robot more human-like behavioral patterns.

Previous research in this field demonstrates a number of effective techniques for creating emotional robots. It has been found that a smooth transition between mood states is crucial for the development of robotic emotions (Stock-Homburg, 2022). The engagement activity of the robots and the user's perception of the robot are both directly influenced by the robot's emotional shift from one mood to another. The empathy of a robot must still be shown through responsive interaction actions. A fixed one-to-one link between the emotional state of a robot and its response is inappropriate. The shift between mood states would be more intriguing and realistic if the robot's expression remained constant. In order to create truly sociable robots, Rincon et al. (2019) developed a social robot that aims to assist older people in their daily activities while also being able to perceive and display emotions in a human-like way. The robot is currently being tested in a daycare center in the northern region of Portugal. Shao et al. (2020) proposed a novel affect elicitation and detection method for social robots in HRIs, which used non-verbal emotional behaviors of the robot to elicit user affect and directly measure it through EEG signals. The study conducted experiments with younger and older adults to evaluate the affect elicitation technique and compare two affect detection models utilizing multilayer perceptron neural networks (NNs) and support vector machines (SVMs).

Rather than being established randomly, the correlations between the emotive response of a robot and its emotional state may be modeled from emotional analysis and used to develop patterns of interaction in the creation of communicative behaviors (Han M. J. et al., 2012).

#### 4.2.6. Multi-modal feedback

Robots can use a combination of multiple modalities to provide feedback, for example, using speech synthesis and visual feedback to indicate status. Multi-modal feedback is a key aspect of multimodal human–robot interaction (HRI), as it allows robots to convey information or commands to the human user through multiple modalities simultaneously (Andronas et al., 2021). This can provide a more comprehensive and engaging user experience. Here are a few ways that multi-modal feedback can be used in HRI.

Multi-modal status indication: Robots can use a combination of multiple modalities such as audio cues and visual cues, to indicate the status of the robot, such as a beep sound and a flashing light when the robot is ready to receive commands, and a different sound and light when it has completed a task.

Multi-modal error indication: Robots can use a combination of multiple modalities, such as a warning tone and a flashing light, to indicate an error or problem with the robot.

Multi-modal cues and prompts: Robots can use a combination of modalities such as speech synthesis, visual cues and sound to prompt the user to perform a specific action, this can make the instruction clear and easy to follow. For example, a robot assistant in a factory might use a combination of a flashing light and speech synthesis to prompt the user to perform a specific task (Cherubini et al., 2019).

Multi-modal social presence: Robots can use a combination of modalities such as speech synthesis, facial expressions and sound effects to create a sense of social presence and make the robot more relatable and human-like.

Multi-modal information: Robots can use a combination of modalities such as speech synthesis, visual cues, and haptic feedback to convey information, this can make the information more intuitive and easy to understand. For example, a robot designed to provide directions might use a combination of speech synthesis and visual cues to display a map and provide turn-by-turn directions.

Multi-modal dialogue management: Robots can use a combination of modalities such as speech recognition, computer vision, and haptic feedback to manage the dialogue between the human and the robot, this can allow for more seamless and natural interactions. For example, a robot assistant in a hospital might use speech recognition to understand the user's request, computer vision to locate the necessary supplies, and haptic feedback to alert the user when the supplies have been retrieved (Ahn et al., 2019).

### 4.3. Application of multimodal HRI

**Figure 6:**
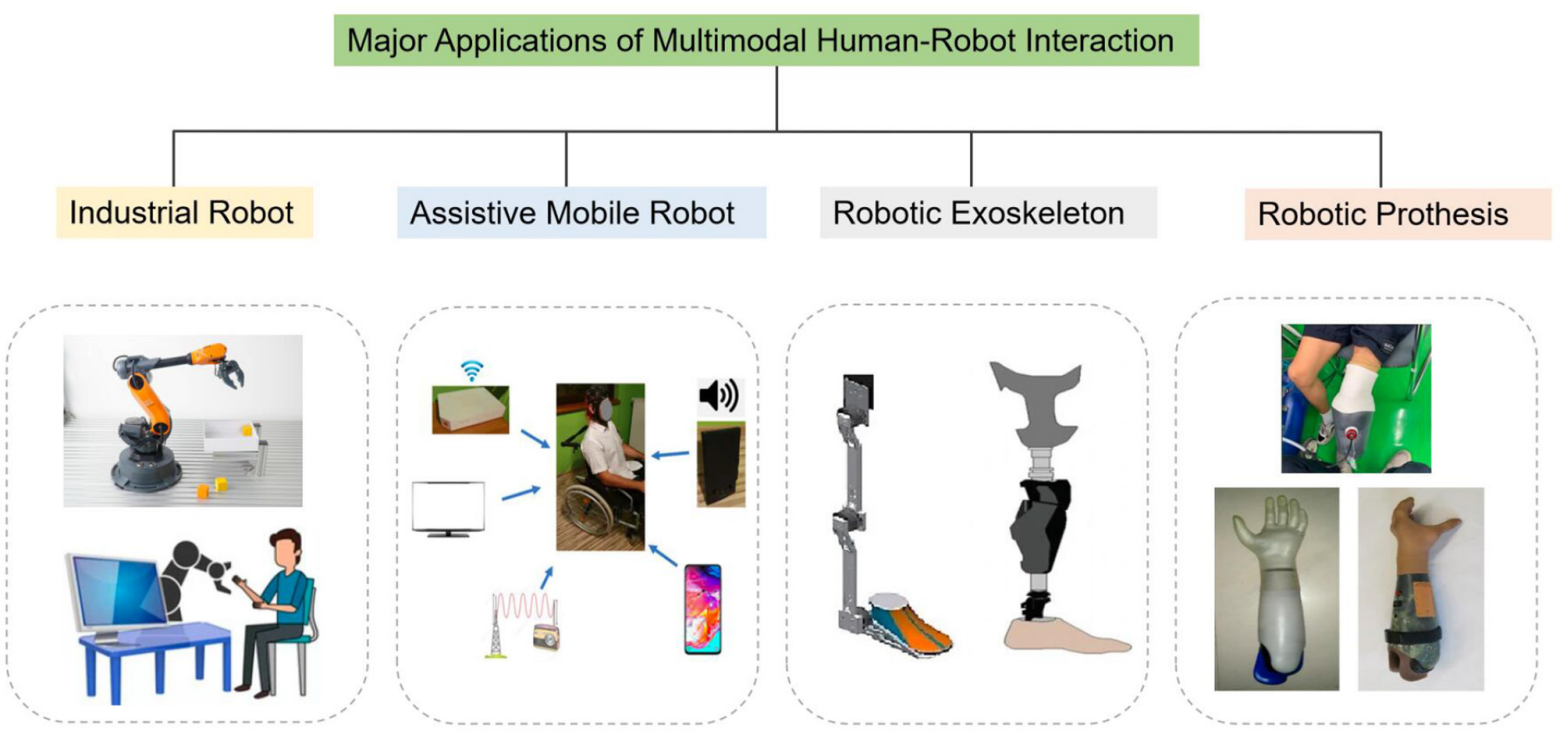
Major applications of multimodal human–robot interaction (Hahne et al., 2020; Kavalieros et al., 2022; Mocan et al., 2022; Pawuś and Paszkiel, 2022; Sasaki et al., 2022).

With the fast advancement in sensors and HRI innovations, numerous applications of multimodal HRI have sprung up in recent years. In this section, four major applications will be briefly introduced, that is, industrial robots, assistive mobile robots, robotic exoskeletons, as well as robotic prothesis, as shown in Figure 6.

#### 4.3.1. Industrial robots

In the last few years, there has been a huge leap in the productivity and marketability of industrial robots, and the use of industrial cobots has significantly aided in the growth of the industry. Industrial cobots, or collaborative robots, are designed to work alongside human operators in various tasks and environments. As a result of the development and popularity of Industry 4.0, industrial cobots are now expected to be increasingly independent and smart to complete more complicated and flexible jobs. Industrial robot growth is dependent on the development of several technologies, of which sensing technologies are a crucial component. Sensors can be employed to gather a wealth of data to assist industrial cobots in carrying out their duties, that is to say, industrial cobots need sensors to carry out their functions.

There are four kinds of sensors used on industrial robots: visual sensors, tactile sensors, laser sensors, and encoders (Li and Liu, 2019). Apart from the four types of sensors, other sensors used in industrial cobots to perform various activities include proximity sensors, ultrasonic sensors, torque sensors, inertial sensors, acoustic sensors, magnetic sensors, and so on.

Sensors are widely employed to aid users in controlling industrial cobots to perform assigned activities that include human–robot collaboration (HRC), adaptive cruise control, manipulator control, and so on. The notion of HRC was recently introduced to actualize the joint operation of employees and robots. This form of production can increase the flexibility and agility of manufacturing systems by combining human cognition and strain capacity with the precision and tirelessness of robots. The fundamental issue that HRC must address is safety. Robotic machines should be capable of detecting and recognizing things in order to prevent conflicts or to stop movement instantly in the event of a collision. Vision sensors, proximity sensors, laser sensors, torque sensors, and tactile sensors are popular sensors used to execute this function. For example, in this paper (O'Neill et al., 2015; Fritzsche et al., 2016), tactile sensors are used to detect physical touch and pinpoint the location of accidents in order to protect personnel who are collaborating with the robot. In Popov et al. (2017), inner joint torque sensors are used to identify and categorize collisions by calculating external forces.

Interaction between robots and humans can be crucial in human–robot collaboration. Workers can successfully control computer programming via HRI. In Kurian (2014), for example, voice recognition supported by acoustical sensors is utilized to assist people in interacting with robots. However, if the surrounding environment is noisy, such a method may not work well. To address this issue, hand gesture identification using vision sensors has been presented in Tang et al. (2015). The integration of multiple sensor types and technologies allows cobots to adapt to various working conditions and enhances the efficiency and safety of human–robot collaboration in industrial settings.

#### 4.3.2. Assistive mobile robots

For more than two decades, researchers have been adapting mobile robotic principles to assistance devices, which corresponds to two key applications: intelligent wheelchairs and assistive walkers. The smart wheelchair is among the most commonly used assistive equipment, with an estimated user base of 65 million globally. Wheelchairs can be either manual or power-driven. Many wheelchair users find it difficult to utilize their wheelchairs autonomously due to the lack of skills, muscles, or vision. An intelligent wheelchair is simply a motorized wheelchair outfitted with sensors plus digital control systems.

The advancement of navigation algorithms for obstacle detection, automated user transportation, and aided steering of the wheel through cutting-edge human–robot interfaces are all outcomes of studies on intelligent wheelchairs. The idea that the regularly used joysticks are not always helpful, especially for users with a low degree of neuro-muscular competence, is the primary driving force behind these studies. Smart wheelchairs include a variety of sensors, including cameras, infrared, lasers, and ultrasonic (Desai et al., 2017). Modern technologies have increasingly been incorporated into user interfaces in an attempt to enhance user independence or entirely automate the product's navigation.

Touch screens, voice recognition systems, and aided joysticks are employed to transmit the locations or routes to the robotic machine (Schwesinger et al., 2017). Some research projects focused on creating frameworks that let users or the controller receive force input from the surroundings using haptic interface for individuals with vision problems (Chuy et al., 2019). Other more recent methods used speech commands and audible feedback to communicate choices to the operator, such as how to navigate around obstacles, safely approach items, and reach items from a certain angle (Sharifuddin et al., 2019). Many advancements have been made to convert users' eye, facial, and body motions into orders for the wheelchair using the visual output for sufferers who are unable to handle a normal joystick (Rabhi et al., 2018a,b). The same method of classifying and recognizing gestures is used to collect surface EMG (Kumar et al., 2019) and EEG signals (Zgallai et al., 2019). Some research projects that employ many input sources, including biosignals and feedback sensors, in tandem to conduct aided navigation also examine multimodal sensory integration (Reis et al., 2015). For aided navigation and steering of walkers, ML algorithms are combined with sensors and bio-signal gathering frameworks, comparable to the HRI techniques for intelligent wheelchairs (Alves et al., 2016; Caetano et al., 2016; Wachaja et al., 2017). These intelligent walkers may be used by individuals with visual impairments for secure outdoor and indoor activity.

#### 4.3.3. Robotic exoskeletons

A robotic exoskeleton serves as an active orthosis device that should be transportable in everyday life situations to assist patients with movement and control limitations (der Loos et al., 2016). Furthermore, robotic exoskeletons might be a feasible option for industrial cargo bearing, as industrial personnel do repetitive physical duties exposing them to musculoskeletal problems (Treussart et al., 2020). An essential component in the control of robotic exoskeletons is the acquisition and identification of human intent, which is carried out by different means of human–robot interaction and acts as an input to the control system.

Cognitive human–robot interaction (cHRI) utilizes EEG signals from the central nervous system to the musculoskeletal system, or surface EMG signals, to recognize the client's needs prior to any real body movements and then estimate the appropriate torque or positional inputs. When compared with the lower-limb exoskeletons, research activities on upper-limb exoskeletons concentrate on developing interface and decoding methodologies to enable accuracy and agility in a larger range of motions. ML techniques are very effective for recognizing the user's mobility intentions grounded in categorized biological signals and may be used to operate such equipment in live time (Nagahanumaiah, 2022).

Physical human–robot interaction (pHRI) employs force measures or alterations in joint locations caused by musculoskeletal system movement as control inputs to the robotic exoskeleton. In such circumstances, the robot's controller seeks to minimize the effort required to complete the tasks, resulting in compliant action. To be more precise, in HRI, minimal contact pressures are preferred and task-tracking mistakes should be avoided. To achieve this, interacting forces are usually controlled using resistance or admission controllers that employ a virtual impedance term to simulate HRI, as described by Hogan (1984).

#### 4.3.4. Robotic prothesis

A robotic prosthetic limb is a robot that is linked to a sufferer's body and replicates its capabilities in everyday routines (Lawson et al., 2014). Robotic prostheses come into direct contact with the body since their functions are often controlled and directed in real-time by clients by muscular or cerebral impulses. Physical specifications, an anthropomorphic appearance, deciphering user's intention, and replicating movements, force efforts, or grip shapes of the actual body are all key characteristics to consider while constructing a robotic prosthesis. Many recent studies have concentrated on producing prosthetics that more nearly resemble the abilities of a lost organic limb (Masteller et al., 2021). The key to effective growth is to acquire a precise technique of recognizing the client's needs, ultimately, perceiving the surroundings and transforming that need into action. The mobility and dynamic control systems of the robot may capture a mixture of bio-signals to construct an identification scheme and actualize the user intention. These data are biometric records of residual limb muscular electrical activity, brain function, or contact stress in sockets. The prosthesis's control system gets complicated input patterns from the client and makes real-time motor control decisions based on the learnt forecast of the user's purpose. Pattern recognition technologies applied to myoelectric or other biosignals are used to identify user-intentioned behaviors. Typically, a classifier is taught to distinguish various robotic prosthesis joint actuators using patterns from multi-channel EMG data.

In order to strengthen the resilience of the activities performed by the equipment, the study on this topic is primarily focused on enhancing the understanding of myoelectric patterns and the concurrent pattern identification and management of numerous functions. Essentially, assignments from everyday routines have many degrees of freedom to move simultaneously. Hence, integrated joint movements must be categorized differently. Deep learning (DL) techniques have recently been used as a novel tool to conduct classification and regression tasks straight from high-dimensional raw EMG signals without locating and recognizing any signal characteristics (Ameri et al., 2018).

## 5. Recent advancements of application for multi-modal human–robot interaction

Multimodal HRI has advanced greatly in the past decade, with numerous research progress and applications coming into existence each year. In this section, we systematically review the state of the art of multimodal HRI, and thoroughly comb the research progress in terms of the signal input, the signal output, as well as the applications of multimodal HRI by listing and summarizing relevant articles.

### 5.1. Multimodal input for human–robot interaction

This section presents a systematic literature review summarizing the latest research progress in signal input for multimodal human–robot interaction (HRI).

#### 5.1.1. Intuitive user interfaces and multimodal interfaces

In recent years, numerous studies have been conducted to improve signal input in HRI. Salem et al. (2010) describe a method for enabling the bipedal robot ASIMO to generate voice and co-verbal gestures freely at runtime without being constrained to a predefined repertoire of motor motions. Berg and Lu (2020) summarize research methodologies on HRI in service and industrial robotics, emphasizing that advancements in human–robot interfaces have brought us closer to intuitive user interfaces, particularly when adopting multimodal interfaces that include voice and gesture detection.

#### 5.1.2. Audiovisual UI and multimodal feeling identification

Ince et al. (2021) present research on an audiovisual UI-based drumming platform for multimodal HRI, creating an audiovisual communicative interface by combining communicative multimodal drumming with humanoid robots. Chen et al. (2021) explore multimodal emotion identification and intent understanding, presenting various modalities of mental feature extraction and emotion recognition methods, and applying them in practice to achieve HRI.

#### 5.1.3. Natural interaction framework and seamless communication

Andronas et al. aim to develop and implement a natural interaction framework for human-system and system-human communication, allowing seamless communication between controllers and “robot companions.” An automobile sector scenario evaluates the framework's performance, showing how an intuitive interface framework can enhance the effectiveness of both humans and robots (Andronas et al., 2021).

#### 5.1.4. Nonverbal communication, locomotion training, emotional messaging, and multimodal robotic UI

Various techniques and systems have been explored to improve human–robot interaction through nonverbal communication, locomotion training, emotional messaging, and multimodal robotic UI. Han J. et al. (2012) introduce a novel method to investigate the application of nonverbal signals in HRI using the Nao system, which includes an array of sensors, controllers, and interfaces. The findings suggest that individuals are more inclined to interact with a robot that can understand and communicate through nonverbal channels.

#### 5.1.5. Active engagement and multimodal HRI solutions

Gui et al. develops a locomotion trainer with multiple walking patterns that can be regulated by participants' active movement intent. A multimodal HRI solution, including cHRI and pHRI, is designed to enhance subjects' active engagement during therapy (Gui et al., 2017). Additionally, a MEC-HRI system featuring various emotional messaging channels, such as voice, gesture, and expression, is presented. The robots in the MEC-HRI platform can understand human emotions and respond accordingly (Liu et al., 2016).

#### 5.1.6. Spatial language and multimodal robotic UI

Research on robot spatial relations employs a multimodal robotic UI. They demonstrate how to extract other geographical information, such as linguistic geographical descriptions, from the evidence grid map. Examples of spatial language are provided for both human-to-robot input and robot-to-human output (Skubic et al., 2004). It can be said without doubt that signal input is an indispensable part of human–robot interaction.

#### 5.1.7. Prosody cues, tactile communication, and proxemics computational method

Recently, significant progress has been made in the field of multimodal HRI, with signal recognition becoming a hot topic. Aly and Tapus investigate the relationship between nonverbal and para-verbal interaction by connecting prosody cues to arm motions. Their method for synthesizing arm gestures employs coupled hidden Markov models, which can be thought of as a cluster of HMMs representing the streams of divided prosodic qualities and segmented rotational features of the two arms' expressions (Aly and Tapus, 2012). Tactile communication might be used in multimodal communication networks for HRI. Two studies were carried out to evaluate the viability of employing a vocabulary of standard tactons within a phrase for robot-to-human interaction in tactile speech (Barber et al., 2015).

#### 5.1.8. Collaborative data extraction and computational framework of proxemics

Whitney et al. (2017) offer a model that relies on agent collaboration to achieve richer data extraction from observations. This paper proposes a mathematical formulation for an item-fetching area that enables a robot to improve the speed and precision with which it interprets a person's demands by speculating about its own ambiguity and processing implicit messages. Mead and Mataric (2012) present a computational framework of proxemics based on data-driven probabilistic models of how social signals (speech and gestures) are produced by a human and perceived by a robot. The framework and models were implemented as autonomous proxemic behavior systems for sociable robots.

#### 5.1.9. Latest advancements in input signals for multimodal HRI

The following studies highlight the latest advancements in input signals used to improve multimodal human–robot interaction. Alghowinem et al. (2021) provide a proxemics computational method based on info-driven probabilistic models of how humans make and robots receive social signals, including gestures and speech. The structure and models were applied as sociable robots' autonomous proxemic behavior systems. Tuli et al. (2021) provide a notion for semantic visualization of human activities and intent forecast in a domain knowledge-based semantic info hub utilizing a flexible task ontology interface. Bolotnikova (2021) study the subject of whole-body anthropomorphic robot postural planning in the setting of assistive physical HRI. They extend the non-linear optimization-based stance-generating system with the elements required to design a robot stance in communication with a human point cloud.

#### 5.1.10. Multimodal features and routine development

Barricelli et al. (2022) provide a fresh method for routine development that takes advantage of Amazon Alexa on Echo Show devices' multimodal characteristics (sight, voice, and touch). It then shows how the suggested technique makes it easier for end users to construct routines than the traditional engagement with the Alexa app.

The articles listed above have summarized the latest application of input signals used in multimodal HRI.

### 5.2. Multimodal output for human–robot interaction

This section follows the recent research development in multimodal HRI signal input by presenting a comprehensive literature review and identifying related studies in this field.

As robots become increasingly sophisticated, they are capable of generating multimodal signals, drawing the attention of researchers in the field. Gao et al. (2021) present a strategy based on multimodal information fusion and multiscale parallel CNN to increase the precision and validity of hand gesture identification. Yongda et al. (2018) describe a multimodal HRI method that combines voice and gesture, creating a robot control system that converts human speech and gestures into instructions for the robot to perform. Li et al. (2022) present a unique Multimodal Perception Tracker for monitoring speakers using both auditory and visual modalities, leveraging a lens model to map sound signals to a localization space congruent with visual information.

The latest developments in multimodal output for multimodal human–robot interaction include unique emotion identification systems, multimodal conversation handling, and voice and gesture recognition systems for natural interaction with humans. Cid et al. (2015) offer a unique multimodal emotion identification system that relies on visual and aural input processing to assess five different affective states. Stiefelhagen et al. (2007) present systems for recognizing utterances, multimodal conversation handling, and visual processing of a user, including localization, tracking, and recognition of the user, identification of pointing gestures, and recognition of a person's head orientation. Zlatintsi et al. (2018) investigate new aspects of smart HRI by automatically recognizing and validating voice and gestures in a natural interface, providing a thorough structure and resources for a real-world scenario with elderly individuals assisted by an assistive bath robot.

Rodomagoulakis et al. (2016) develop a smart interface featuring multimodal sensory processing abilities for human action detection within the context of assistive robots, exploring cutting-edge techniques for automated-localization cognition and visual activity recognition to multimodally identify commands and activities. Loth et al. (2015) measure the recognizer modes that are important at various levels of human–robot interaction, providing insight into social behavior in humans to create socially adept robots.

In recent years, many contributions have been made to investigate how multimodal signal output influences HRI. Bird (2021) explore ways to give a robot social understanding through emotional perception for both verbal and non-verbal interaction, demonstrating how the framework's technology, organizational structure, and interactional examples address several outstanding concerns in the field. Yadav et al. (2021) provide a thorough analysis of various multimodal techniques for motion identification, using different sensors and analytical strategies with methodological fusion methods. Liu et al. (2022) investigate multimodal information-driven robot control for cooperative assembly between humans and robots, creating a human–robot interface free of programming using function blocks to combine multimodal human instructions that precisely activate specified robot control modes.

Khalifa et al. (2022) present a robust framework for face tracking and identification in unrestricted environments, designing their framework based on lightweight CNNs to increase accuracy while preserving real-time capabilities essential for HRI systems. Shenoy et al. (2021) improve the interaction capabilities of Nao humanoid robots by combining detection models for facial expression and speech quality, using the microphone and camera to assess pain and mood in children receiving procedural therapy. Tziafas and Kasaei (2021) introduce a software architecture that isolates a target object from a congested scene based on vocal cues from a user, employing a multimodal deep neural net as the system's core for visual grounding. The research proposes the CFBRL-KCCA multimodal material recognition framework for object recognition challenges, demonstrating that the suggested fusion algorithm provides a useful method for material discovery (Wang et al., 2021).

The application of multimodal signal output in HRI has become a fast-growing field; however, there are still many challenges to be addressed. As human–robot interaction continues to be a hot research topic, researchers will undoubtedly explore new methods and solutions to enhance multimodal signal output and improve the overall HRI experience.

### 5.3. Major application areas of multimodal HRI

This section provides a comprehensive review and summarizes recent research advances in the application of multimodal HRI by examining numerous recent publications.

The emergence of sensing technologies and the increasing popularity of robotics have enabled researchers to study multimodal HRI, with numerous papers published each year. Gast et al. (2009) present a novel outline for real-time multimodal information processing, designed for scenarios involving human-human or human–robot interaction and including modules for various output and input signals. Chen et al. (2022) develop a real-time, multi-model HRC scheme using voice and gestures, creating a collection of 16 dynamic gestures for human-to-industrial robot interaction and making a data collection of dynamic gestures publicly available.

Haninger et al. (2022) introduce a unique approach for multimodal pHRI, creating a Gaussian process model for human power in each state of a joint effort, and applying these frameworks to model predictive command and Bayesian inference of the style to forecast robot responses. Thomas et al. (2022) present a multimodal HRI platform that combines speech and hand sign input to control a UGV, translating vocal instructions into the ROS environment to drive the Argo Atlas J8 UGV using Mycroft, an accessible digital assistant. Švec et al. (2022) introduces a multimodal cloud-based system for HRI, with the key contribution being the construction of the architecture based on industry-recognized frameworks, protocols, and JSON messages that have been verified.

A self-tuning multimodal fusion method is proposed to address the issue of helping robots achieve better intention comprehension. This method is not constrained by the manifestations of interacting individuals and surroundings, making it applicable to diverse platforms (Hou et al., 2022). Weerakoon et al. (2022) present the COSM2IC system, which uses a compact Task Complexity Predictor and multiple sensor data input to evaluate the instructional richness to reduce loss in precision. This structure dynamically switches between a collection of models with different computational intensities so that computationally less demanding models are instantiated whenever viable.

Jooyeun Ham et al. introduce a versatile and elastic multimodal sensor system coupled with a soft bionic arm. They employ a manufacturing strategy that uses both UV laser metallic ablation and plastic cutting concurrently to construct sensor electrode designs and elastic conducting wires in a Kirigami pattern, implementing the layout of wired sensors on an adjustable metalized film (Bao et al., 2022). Bucker et al. (2022) provide a versatile language-based user interface for HRC, taking advantage of recent developments in big language models to encapsulate the operator command, and employing multimodal focus transformers to integrate these characteristics with trajectory data. The mobility signal of the robot and the client's cardiac signal are gathered and combined to provide multimodal data as the input node vector of the DL framework, which is utilized for the control system's model of HRI (Wang W. et al., 2022). Maniscalco et al. (2022) evaluate and suitably filter all the robotic sensory data required to fulfill their interaction model, paying careful attention to backchannel interaction, making it bilateral and visible through audio and visual cues. Wang R. et al. (2022) offer Husformer, a multimodal transformer architecture for multimodal human condition identification, suggesting the use of cross-modal transformers, which motivate one signal to strengthen itself by directly responding to latent relevancy disclosed in other signals. The focus on multimodal HRI has brought many concepts into practice.

Multimodal HRI has been developing rapidly, with numerous new methods for HRI using different modalities being proposed. Strazdas et al. (2022) create and test a novel multimodal scheme for non-contact human-machine interaction based on voice, face, and gesture detection, assessing the user experience and communication efficiency of their current scheme in a large study with many participants. Zeng and Luo suggest a solution for enhancing the precision of multimodal haptic signal detection by improving the SVM multi-classifier using a binary tree. The modified particle swarm clustered technique is utilized to optimize the binary tree structure, minimize the error piling of the binary leaf node SVM multi-classifier, and increase multimodal haptic signal identification accuracy (Zeng and Luo, 2022). Nagahanumaiah (2022) develops a tiredness detection algorithm based on real-time information collected from wearable sensors, with the goal of understanding more about how humans feel fatigued in a supervisory human-machine setting, examining machine learning techniques for tiredness identification, and employing robots to modify their interactions.

Schreiter et al. (2022) aim to deliver high-quality tracking data from activity capture, eye-gaze trackers, and robotic sensors in a semantically rich context, using loosely scripted tasks to produce natural behavior in the videotaped participants, which leads the attendees to move through the changing lab setting in a natural and deliberate manner. In an HRC scenario, Armleder et al. (2022) develop and implement a control scheme that can enable the implementation of large-scale robotic skin, demonstrating how entire tactile feedback may enhance robot abilities during dynamic interplay by delivering information about various contacts throughout the robot's exterior.

The application of multimodal HRI is extensive, including using multiple sensors and inputs to evaluate social interactions, incorporating time delay and context data to improve recognition and emotional depiction, and developing unique models that combine different modalities. Tatarian et al. (2022) provide a multimodal interaction that focuses on proxemics of interpersonal navigating, gaze mechanics, kinesics, and social conversation, examining the impact of multimodal actions on relative social IQ using both subjective and objective assessments in a seven-minute encounter with 105 participants. Moroto et al. (2022) develop a recognition approach that considers the time delay to get genuinely near the reality of the occurring mechanism of feelings, with experimental findings demonstrating the usefulness of taking into account the time lag between gazing and brain function data.

He et al. (2022) present a unique multimodal M2NN model using the merging of EEG and fNIRS inputs to increase the recognition speed and generalization capacity of MI, combining spatial-temporal extraction of features, multimodal feature synthesis, and MTL. Zhang et al. (2022) retrieve effective active parts from sEMG data acquired by the MYO wristband using active element detection, then extracting five time-domain parameters from the main section signal: the root average square value, wave duration, number of zero-crossing spots, mean absolute value, and maximum-minimum value. Yang et al. incorporate context data into the current speech by embedding prior statements between interlocutors, which improves the emotional depiction of the present utterance. The suggested cross-modal converter module then focuses on the interconnections between text and auditory modalities, adaptively fostering modality fusion (Yang et al., 2022). Based on the proposed papers listed above, it is clear that multimodality currently plays a significant role in HRI research.

In conclusion, multimodal HRI has seen rapid development and a wide range of applications in recent years. Researchers are exploring various methods and techniques to improve human–robot interaction by using multiple modalities, such as voice, gestures, and facial expressions. As more advancements are made in this field, it is expected that multimodal HRI will continue to play a crucial role in shaping the future of human–robot interaction. The application of multimodal HRI has expanded across various fields, including robotics, healthcare, COVID-19 diagnosis, secure planning/control, and co-adaptation. Researchers have explored the use of multiple modalities in emotion recognition, gesture recognition, EEG and fNIRS data merging, sensor data processing, speech recognition, and human mobility assessment. Additionally, multimodal HRI has shown potential in medical diagnosis and prognosis, such as epilepsy, creating robots with advanced multimodal mobility, AI-aided fashion design, and the integration of robotics and neuroscience.

The advantages of multimodal HRI include natural and intuitive interaction between humans and robots, increased accuracy and robustness in sensing and control, and the ability to handle complex tasks and situations. However, challenges remain, such as data fusion, algorithm development, and system integration.

Multimodal HRI is a growing field with many areas yet to be explored. As research continues, it is expected that multimodal HRI will play a crucial role in shaping the future of human–robot interaction, leading to more efficient, user-friendly, and versatile robotic systems.

## 6. Discussion

Multimodal human–robot interaction is a field of research that aims to improve the way humans and robots communicate with each other. It is based on the idea that humans use multiple modalities, such as speech, gesture, and facial expression, to convey meaning and that robots should be able to understand and respond to these modalities in a natural and intuitive way.

### 6.1. Natural language processing and computer vision

Natural language processing is widely used in multimodal HRI for speech recognition and understanding, which has the advantage of being able to handle a wide range of spoken languages. However, NLP's accuracy and performance are heavily dependent on the quality and quantity of the training data, which can be a challenge for rare or dialectal languages. Moreover, the recognition of ambiguous phrases or slang can lead to incorrect interpretations.

Computer vision techniques, such as gesture and facial expression recognition, have shown great potential in enhancing the naturalness and expressiveness of robot interactions. These techniques can detect subtle and nuanced movements that may be difficult for humans to perceive. However, limitations of computer vision include its sensitivity to lighting conditions, occlusions, and variations in appearance across individuals. Furthermore, these techniques require high computational power, making them unsuitable for resource-constrained robots.

### 6.2. Machine learning and haptic feedback

Machine learning techniques are essential for integrating and interpreting different modalities, including speech, vision, and haptic feedback. ML algorithms enable the robot to recognize and understand complex patterns in multimodal data, making it possible to provide natural and adaptive interactions. However, models may be biased or fail to generalize to unseen data, leading to reduced performance in real-world scenarios.

Haptic feedback and motion planning techniques are particularly useful for physical interaction between humans and robots. Haptic feedback provides a sense of touch, allowing robots to respond to human gestures and movements in a natural way. Motion planning algorithms enable the robot to navigate in a human environment safely and efficiently. However, haptic feedback and motion planning require high precision and accuracy, which can be challenging to achieve in complex and dynamic environments.

### 6.3. Deep learning and touch-based interaction

Although deep learning techniques have shown great potential in recognizing and interpreting human gestures and expressions, there are still some challenges that need to be addressed. One challenge is the need for a large amount of labeled data to train deep learning models, which can be time-consuming and expensive to obtain. Another challenge is the need for robustness to variations in lighting, background, and appearance of human gestures and expressions. Despite these challenges, deep learning techniques have the potential to significantly improve the accuracy and robustness of gesture and expression recognition in human–robot interaction.

The use of haptic feedback for touch-based interaction has great potential for improving the naturalness and intuitiveness of human–robot communication. However, there are still challenges that need to be addressed, such as the need for high-quality and responsive haptic feedback that can mimic human touch, and the need for effective motion planning algorithms that can ensure safe and efficient interactions between humans and robots. Nevertheless, with the ongoing advancements in haptic technology and motion planning algorithms, it is expected that touch-based interaction will become an increasingly important aspect of multimodal human–robot interaction in the future.

### 6.4. Future directions

In the future, there will be more emphasis on creating more natural and intuitive interaction, as well as improving the robots' ability to understand and respond to human emotions. This will be achieved through the integration of emotion recognition and generation algorithms, making robots more human-like. Another trend will be the use of multi-robot systems, in which multiple robots work together to accomplish a task. This will allow for more complex and efficient interactions between humans and robots.

In addition to the integration of emotion recognition and generation algorithms, there will also be a focus on creating robots that can adapt to individual differences in communication style and preferences. This could be achieved through personalized learning and adaptation techniques. Finally, ethical considerations in human–robot interaction will become increasingly important, and there will be a need for ethical guidelines and regulations to ensure the safe and responsible use of robots in various applications.

## 7. Conclusion

The current state and emerging directions of multimodal human–robot interaction is thoroughly discussed in this paper. Also, we have thoroughly combed the research progress in terms of the information input for multimodal HRI, the information output for multimodal HRI, as well as the concrete applications of multimodal HRI. Specifically, this review elaborates on the research progress of information input for multimodal HRI from three perspectives: gesture recognition, speech recognition, as well as emotion recognition. In terms of information output, gesture generation and emotional expression generation are covered. Research in this area has focused on developing various modalities, such as speech, gesture, and facial expression, to enable robots to understand better and respond to human intentions and emotions. The integration of multiple modalities is also crucial for achieving robust and flexible human–robot interaction. The major limitation of the study lies in the limited number of real-world deployments of multimodal human–robot interaction systems, so the impact of the technology on users may not be well understood. Also, there are technical challenges, such as high computational requirements and system complexity that limit the scalability of multimodal human–robot interaction systems. Hopefully, this paper will reflect the current research trend in human–robot interaction and provide guidance for future research.

## Author contributions

HS and WQ contributed to the draft writing and the other authors contributed to correcting, supervising, and proof reading, etc. All authors contributed to the article and approved the submitted version.
